# De novo Rob translocation 45, XX, rob(13;13)(q10;q10) in a Syrian woman with recurrent miscarriages: A rare case report

**DOI:** 10.1097/MD.0000000000042128

**Published:** 2025-04-18

**Authors:** Joudy Sandouk, Sedra Hamad, Leen Al Sakkal, Marwan Alhalabi

**Affiliations:** a Faculty of Medicine, Damascus University, Damascus, Syria; b Department of Reproductive Medicine, Embryology and Genetics, Faculty of Medicine, Damascus University, Damascus, Syria; c BioTherapeutics Research Center, Faculty of Medicine, Damascus University, Damascus, Syria.

**Keywords:** case report, chromosomal abnormalities, recurrent miscarriage, Robertsonian translocation, uniparental disomy

## Abstract

**Rationale:**

Chromosomal rearrangements play a significant role in 50% of recurrent miscarriage cases. The incidence of chromosomal abnormalities in the population ranges from 1/625 to 1/1500. The 2 most common types of these rearrangements are reciprocal and Robertsonian (Rob) translocation. Rob translocation can be found in 1 in 1000 individuals and is the predominant type in humans. This case highlights the importance of identifying such rare genetic abnormalities in patients with recurrent miscarriage.

**Patient concerns:**

We report a 27-year-old Syrian woman who presented at Orient Hospital in Damascus, Syria, with a history of 7 recurrent miscarriages over 10 years of marriage, 6 of these miscarriages occurred in the first trimester. Additionally, she had 5 failed in vitro fertilization (IVF) attempts. Both the phenotypic appearance and hormonal profiles of the couple were normal. There was no history of recurrent miscarriages among other family members.

**Diagnoses and interventions:**

Chromosomal analysis of the patient using the Giemsa trypsin G-banding (GTG)-banding technique revealed an abnormal karyotype with Rob translocation involving both long arms of chromosomes 13, specifically identified as rob(13;13)(q10;q10).

**Outcomes:**

We provided the patient with comprehensive and precise genetic counseling, ensuring she fully understood the impact of the Rob translocation on her reproductive potential and future family planning.

**Lessons:**

In this case, our results enforce the importance of diagnostic procedures, such as cytogenetic analysis and perinatal counseling, in identifying potential chromosomal rearrangements in couples experiencing recurrent miscarriage. To our knowledge, only one similar case was reported. Further research and case documentation could be used to improve understanding of such rare genetic occurrences.

## 1. Introduction

Recurrent miscarriage is defined as 2 or more consecutive (or 3 or more sporadic) pregnancy losses before 20 weeks of gestational age. It is estimated that 1% to 4% of couples suffer from recurrent miscarriage.^[1]^ Despite the low incidence rate of recurrent miscarriage in the population, for every couple who suffers from recurrent miscarriage, its impact is profoundly significant. Because recurrent miscarriage has numerous physical and psychological effects on women,^[2]^ it is of great importance to put all necessary efforts into finding the etiology that may be treatable in some cases. Only 40% of recurrent miscarriage cases have a definite cause.^[1]^

Many cases of recurrent miscarriage have been associated with chromosomal structural abnormalities, most of which are translocations (reciprocal more than Robertsonian), and fewer are inversions.^[3]^ The most common type of translocation in humans is Rob translocation (ROB).^[4]^ In a ROB, the 2 long arms of 2 acrocentric chromosomes fuse to form one chromosome. ROB translocation can involve any of the human acrocentric chromosomes (13, 14, 15, 21, and 22), resulting in 5 homologous and 10 heterologous ROBs. Although heterologous ROBs can be either inherited from a carrier parent or are much less likely to form de novo, almost all homologous ROBs occur de novo via mitosis.^[5]^

In this paper, we describe a rare case of a de novo balanced ROB with both long arms of chromosomes 13 in a phenotypically normal Syrian female with a history of recurrent miscarriage.

## 2. Case presentation

A nonsmoker couple, aged 27 years (female) and 37 years (male), presented to the fertility clinic at Al Orient Hospital in Syria with a history of 7 recurrent miscarriages throughout their 10-year marriage. Six of these miscarriages occurred in the first trimester, with one occurring in the second trimester. After developing secondary infertility, she underwent 5 cycles of in vitro fertilization (IVF) at different centers, all of which were unsuccessful due to early failure of embryo implantation. There were no documented instances of recurring miscarriages among other relatives within the family. Both individuals appeared phenotypically normal, and the wife had regular menstrual cycles. The wife’s BMI was calculated to be 26.7, indicating that she was overweight. Her hormonal profile was within the normal range, with a basal follicle-stimulating hormone level of 8.7 mmol/mL, a thyroid-stimulating hormone level of 1.3 μIU/L, an estradiol level of 32 pg/mL, and a prolactin level of 17 ng/mL. Hysteroscopy, hysterosalpingography, and pelvic ultrasound imaging were performed, and all the results were normal. For the patient’s husband, the clinical examination and semen analysis results were within normal range. Informed consent was obtained from the couple before writing this report.

## 3. Chromosomal analysis

**Figure 1.**
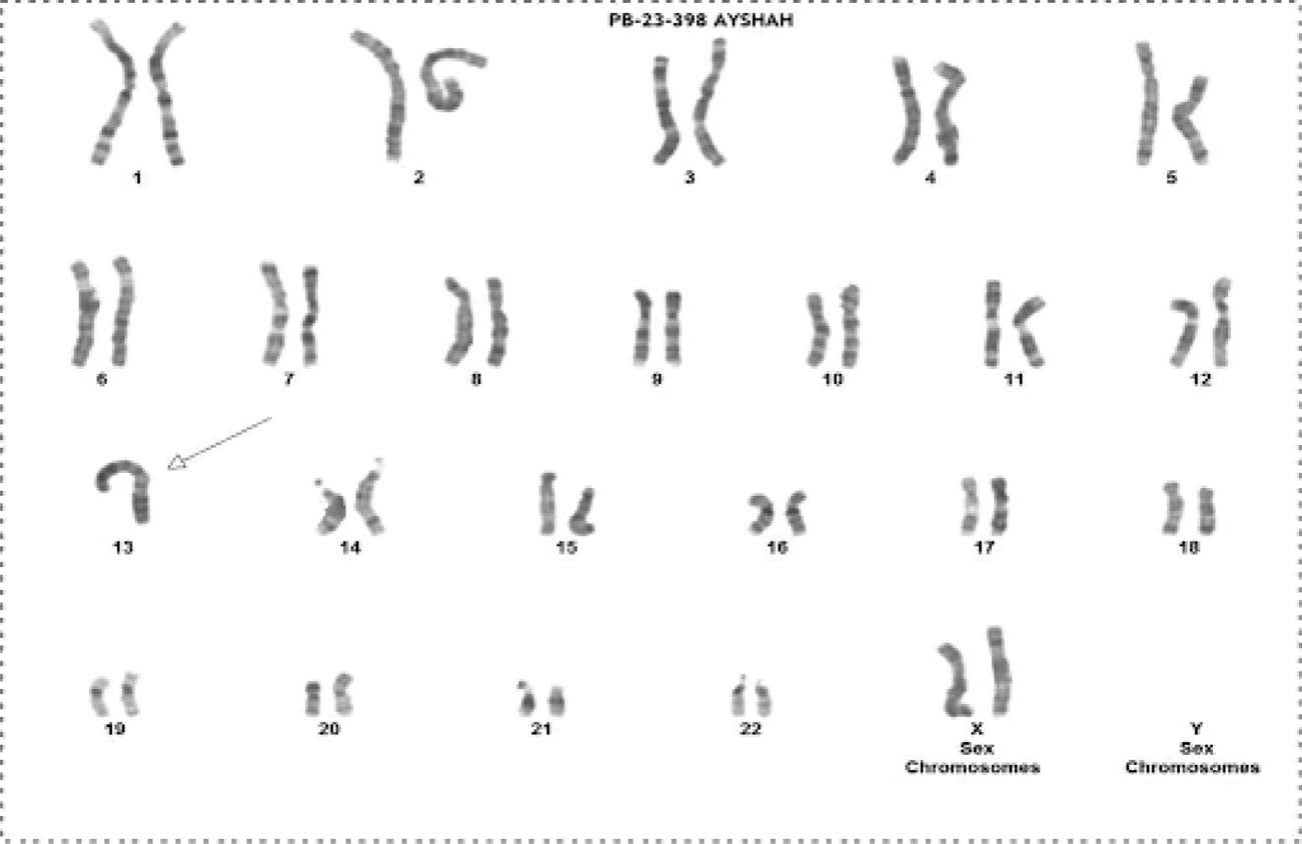
GTG-banding for the patient revealing 45, XX, der(13;13)(q10;q10). The derivative chromosome is marked by an arrow. GTG = Giemsa trypsin G-banding.

Chromosomal analysis by the Giemsa trypsin G-banding (GTG)-banding on the female revealed the presence of a translocation between the long arm of chromosome 13 at band 13q10 and the long arm of chromosome 13 at band 13q10 (Fig. 1). Thus, her karyotype was determined as follows: 45, XX, der(13;13)(q10;q10).

## 4. Discussion

Recurrent miscarriage remains a significant reproductive hurdle for both patients and physicians. Fifteen percent of pregnancies end up with miscarriage. The majority of these miscarriage occur during the first trimester of pregnancy, with 50% attributed to chromosomal abnormalities.^[6]^ Couples facing 2 to 3 recurrent miscarriages are advisable to undergo chromosomal analysis as well as assisted reproductive treatment procedures. Prenatal genetic counseling and IVF with preimplantation genetic diagnosis are 2 examples of treatments, particularly for cases involving chromosomal abnormalities. However, some of these treatments might have potential emotional or financial burdens; therefore, it is essential to base treatment decisions on the underlying causes and associated risk factors.^[7–9]^ A study conducted at the Department of Medical Biology and Genetics in Turkey included 645 couples (1290 patients) with recurrent miscarriages, fetal abnormalities, and/or fetal death. Translocations were shown in 16 patients, 7 of whom had ROBs (4 women carriers vs 3 men carriers).^[10]^ Chromosomal rearrangements can be found in the general population from 1/625 to 1/1500. Among these, ROBs are the most prevalent, occurring in approximately 1 in 1000 individuals. ROBs arise as a result of the fusion of 2 acrocentric chromosomes at their centromeres. The most common types are the nonhomologous forms, which involve 2 different acrocentric chromosomes from distinct pairs, typically chromosomes 13, 14, 15, 21, and 22. Even though any of the 5 human acrocentric chromosomes can be involved in a ROB, the distribution of the various potential translocations in the population remains nonrandom. For instance, almost 85% of all ROBs are rob(13q14q) and rob(14q21q), and only 10% are homologous. In our case, the ROB translocation exhibited a homologous form, representing an uncommon occurrence. Meaning, that the involved chromosomes are from the same pair, which in our case are chromosomes 13.^[4,11,12]^ Recent papers have shown that almost all homologous translocations present as de novo, non-inherited cases. De novo creation’s rate of ROBs is calculated to be approximately 3.9 × 10^–4^ mutations per gamete per generation.

There are 2 possible mechanisms through which homologous ROBs can occur: 1- Within the zygote, as a result of the fusion of 2 homologous acrocentric chromosomes, 1 from each parental gamete. 2- In a monosomic gamete that contains an isochromosome due to nondisjunction, followed by its reduplication, a chromosome that has 2 copies of a single parental acrocentric is generated. This process is referred to as uniparental disomy (UPD). The occurrence rate of UPD on specific chromosomes in live births is estimated to be 1/3500. Chromosomes 13 and 15 are great examples of chromosomes that have specific regions in which they are more likely to have negative effects, although not all UPD events are harmful.^[4,13]^

A retrospective review of 16,749 pregnant women who underwent amniocentesis between January 1981 and December 2010 at a single tertiary medical center in Taiwan revealed that ROBs occurred in 39 cases, 17 of which were de novo or approximately 43% of all ROB cases. Furthermore, an investigation revealed that the most common *de novo* types involve chromosomes 13 and 14.^[14]^

According to a thorough literature review, a similar case was found in Turkey. Serap et al^[15]^ described a case of rob(13;13)(q10;10q) in a woman with recurrent miscarriage. The karyotype was determined to be 45, XX, rob(13;13)(q10;q10) and additional karyotypes for the patient’s husband and parents were normal.

Usually, no phenotypic effect will appear in the carrier of a balanced ROB, as our patient. However, during gametogenesis, it may either inhibit meiosis within the carrier, leading to abnormalities in the offspring or lead to malsegregation of the carrier’s chromosomes. In other words, homologous ROB carriers can only produce unbalanced gametes (either disomic or nullisomic), leading to a fertilized ovum with chromosomal abnormalities-either trisomy or monosomy of one of the chromosomes involved in the translocation. This in turn may lead to recurrent miscarriage or abnormal live birth. Infrequently, a phenotypically normal child can be conceived by a homologous ROB carrier parent if the free chromosome from the unaffected gamete is eliminated at early mitosis via a postzygotic trisomy rescue mechanism.^[5,16]^

## 5. Conclusion

In summary, couples with recurrent miscarriages are encouraged to undergo cytogenetic analysis, before starting the path of infertility treatments, to ensure that they do not have any chromosomal abnormalities that would make assisted reproductive methods fail. In our patient, rob(13;13)(q10;q10) was the reason for recurrent miscarriages. Surrogacy, gamete donation, and adoption are methods that offer alternative paths to parenthood despite biological limitations. Many considerations need to be taken into account regarding these methods, including legal regulations and religious beliefs.

## Acknowledgments

We are grateful to the Faculty of Medicine of Damascus University for their support.

## Author contributions

**Conceptualization:** Joudy Sandouk, Sedra Hamad, Leen Al Sakkal.

**Investigation:** Joudy Sandouk, Sedra Hamad, Leen Al Sakkal.

**Project administration:** Joudy Sandouk, Sedra Hamad, Leen Al Sakkal.

**Resources:** Joudy Sandouk, Sedra Hamad, Leen Al Sakkal.

**Supervision:** Marwan Alhalabi.

**Writing – original draft:** Joudy Sandouk, Sedra Hamad, Leen Al Sakkal.

**Writing – review & editing:** Joudy Sandouk, Sedra Hamad, Leen Al Sakkal.
